# Current View on Osteogenic Differentiation Potential of Mesenchymal Stromal Cells Derived from Placental Tissues

**DOI:** 10.1007/s12015-014-9569-1

**Published:** 2014-11-09

**Authors:** Gabriela Kmiecik, Valentina Spoldi, Antonietta Silini, Ornella Parolini

**Affiliations:** Centro di Ricerca E. Menni, Fondazione Poliambulanza-Istituto Ospedaliero, Via Bissolati, 57, 25124 Brescia, Italy

**Keywords:** Human placenta, Mesenchymal stromal cells (MSC), Amniotic membrane, Chorionic membrane, Umbilical cord, Wharton’s jelly, Decidua, Differentiation, Osteogenesis

## Abstract

Mesenchymal stromal cells (MSC) isolated from human term placental tissues possess unique characteristics, including their peculiar immunomodulatory properties and their multilineage differentiation potential. The osteogenic differentiation capacity of placental MSC has been widely disputed, and continues to be an issue of debate. This review will briefly discuss the different MSC populations which can be obtained from different regions of human term placenta, along with their unique properties, focusing specifically on their osteogenic differentiation potential. We will present the strategies used to enhance osteogenic differentiation potential in vitro, such as through the selection of subpopulations more prone to differentiate, the modification of the components of osteo-inductive medium, and even mechanical stimulation. Accordingly, the applications of three-dimensional environments in vitro and in vivo, such as non-synthetic, polymer-based, and ceramic scaffolds, will also be discussed, along with results obtained from pre-clinical studies of placental MSC for the regeneration of bone defects and treatment of bone-related diseases.

Mesenchymal stromal cells (MSC) were described within the bone marrow (BM) in 1968 by Friedenstein and colleagues [1]. Since then, many other sources have been identified such as adipose tissue [*reviewed in* [2, 3]], cord blood [*reviewed in* [4]], and one which has recently attracted much attention is human term placenta [*reviewed in* [5]], mainly for its easy, non-invasive procurement and abundant tissue, and also for the remarkable immunomodulatory capabilities of MSC isolated from placental tissues. It is well known that the different sources of MSC present different capabilities to differentiate toward mature lineages [6]. This review will focus on data reported thus far regarding the osteogenic differentiation potential of mesenchymal stromal cells deriving from placental tissues.

## MSC Derived from Different Placental Regions

Human placenta is composed of a fetal part, including the amnion, chorion, umbilical cord, and a maternal part, termed decidua. Within these components different cell subpopulations with mesenchymal characteristics may be isolated, and as established by the consensus of the First International Workshop on Placenta-Derived Stem Cells, they are referred to amniotic mesenchymal stromal cells (hAMSC) and chorion mesenchymal stromal cells (hCMSC) [5]. MSC can also be isolated from umbilical cord (UC) and have been referred to as either hUC-MSC or Wharton’s jelly (hWJ)-MSC [7], and from the maternal decidua (hDMSC) [8]. Commonly used methods for MSC isolation are enzymatic digestion employing collagenase and DNase, dispase, trypsin, or explant culture [5, 9]. In general, placental tissues-derived mesenchymal stromal cells as MSC from other sources present spindle-shape, elongated morphology, adherence to plastic and CFU capacity [10].

MSC derived from different regions of human placenta, similar to BM-MSC, express typical mesenchymal antigens such as CD90, CD73, CD105, CD13, CD44, CD29, CD166, CD117, HLA-A, −B, −C, and lack hematopoietic markers CD34, CD45, CD14, endothelial marker CD31, co-stimulatory molecules CD80, CD86, CD40, CD40L, and HLA-DR [11–14]. Some reports have suggested the presence of pluripotent markers Sox-2, OCT-4, Nanog, and SSEA-4 in placental MSC [8, 15–17]. Differentiation potential toward the mesodermal lineage, and specifically toward the adipogenic, chondrogenic, and osteogenic lineages, has been reported for Wharton’s jelly, decidua, and fetal membrane (FM)-derived MSC [5, 8, 9]. Others have also reported in vitro differentiation potential toward ectodermal (neurogenic), as well as endodermal (hepatogenic) lineage of FM-derived MSC and hWJ-MSC [18–20].

Placental tissue MSC also present low expression of MHC class II and (as mentioned above) classical co-stimulatory molecules, features that have made them to be considered as poor antigen presenting cells [21]. However, caution is warranted considering that the same interpretation has been used for BM-MSC, but previous reports have shown the capability of these cells to induce an immune response [22, 23]. Moreover, it has been reported that MSC derived from different placental tissues can interact with and modulate a variety of immune cells [24]. The ability to suppress lymphocyte proliferation induced by mitogens or alloantigens [25, 26], and the capacity to block maturation of monocytes into dendritic cells [27, 28], are examples of their immunomodulatory effects. Furthermore, immunomodulatory molecules, such as HLA-G, B7-H1 and B7-H3, and prostaglandins, secreted from early and term placenta, which have important implications in the fetal maternal tolerance mechanisms, have been reported to be expressed not only in the trophoblast but also in the FM-derived cells, hWJ-MSC and hDMSC [[29–32] and *reviewed in* [14]] and therefore, very likely play a role in the immunomodulatory capacities of these cells [33].

## In Vitro Osteogenic Potential

As previously stated, mesenchymal cells derived from different placental tissues differentiate toward “classical” mesodermal lineages [5, 9]. Herein, we summarize the results obtained from approximately 150 published papers which investigate the osteogenic differentiation of mesenchymal cells derived from amnion, chorion, umbilical cord, and decidua (Tables 1, 2, and 3). We will use hAMSC, hCMSC, hUC-MSC (or hWJ-MSC), and hDMSC to describe the MSC from specified placental tissues, according to the information provided by the authors, while we will use hPD-MSC to refer to placenta derived-mesenchymal stromal cells in general, and when the specific region of placenta used was not indicated. Since it was not possible to be fully comprehensive in the limited space available, we apologize in advance to authors whose work was not cited in this review.

**Table 1:** Summary of studies describing in vitro osteogenic differentiation of MSC derived from fetal and maternal tissues

	Placental region	Gestation time	MSC lineages used	Passage	Growth support	Readout	References
Fetal	Amnion	Term/NS	Bulk population	P0	Plastic	Cytochemical staining	[114]
				Cultured passages	Plastic	Cytochemical staining	[44, 48, 60, 115–120]
					Plastic	Gene evaluation	[48, 116–118]
					Collagen I coated plastic	Cytochemical staining	[65, 121]
					Collagen I coated plastic	Enzymatic evaluation	[121]
					Collagen I coated plastic	Protein evaluation	[65]
					Nukbone® scaffold	Gene evaluation	[70]
			CD271^+^	P0	Plastic	Cytochemical and immunostaining, gene evaluation	[10]
			CD105^+^	Cultured passages	Plastic	Cytochemical staining	[36]
			CD44^+^/CD73^+^/CD105^+^ and CD44^+^/CD73^+^/CD105^−^		Plastic	Enzymatic and protein evaluation, biochemical assay	[15]
			hTERT transduced cells		Plastic	Cytochemical staining, enzymatic and gene evaluation	[122]
			Side population		Plastic	Cytochemical and immunostaining, enzymatic and gene evaluation	[38]
		1st trim, term	Bulk population		Plastic	Cytochemical and immunostaining, enzymatic evaluation	[18]
		2nd trim, term	Bulk population		Plastic	Cytochemical staining	[123]
	Chorion	Term/NS	Bulk population	Cultured passages	Plastic	Cytochemical staining	[20, 48, 119, 124–129]
					Plastic	Immunostaining	[125]
					Plastic	Enzymatic evaluation	[124, 125]
					Plastic	Gene evaluation	[20, 48, 125, 127, 128]
					Plastic	Biochemical assay	[124]
				P0	PU scaffold	Cytochemical staining	[80]
			CD271^+^	P0	Plastic	Cytochemical and immunostaining, gene evaluation	[10]
			Fibroblast depleted and not-depleted cells	Cultured passages	Plastic	Cytochemical staining, enzymatic evaluation	[130]
			CD105^+^		Plastic	Cytochemical staining	[36]
			Bmi-1 or hTERT + Bmi-1 transduced cells		Plastic	Cytochemical staining	[131]
			CD106^+^ and CD106^−^		Plastic	Cytochemical staining	[39]
			C-kit^+^		Plastic	Immunostaining	[132]
			CD31^−^/CD45^−^		Plastic	Cytochemical staining, enzymatic and gene evaluation	[107]
		1st trim	Bulk population	Cultured passages	Plastic	Cytochemical staining	[112, 133, 134]
			Bulk population		Plastic	Enzymatic evaluation	[133]
		1st trim, term	Bulk population		Plastic	Cytochemical staining	[18, 111, 135]
Fetal	Chorion	1st trim, term	Bulk population	1st trim, term	Plastic	Immunostaining, enzymatic evaluation	[18]
			Bulk population		Plastic	Gene and protein evaluation	[111]
			Bulk population		Cell-free chorion scaffold	Cytochemical and immunostaining, enzymatic and gene evaluation, electron microscopy	[71]
Maternal	Decidua	Term	Bulk population	Cultured passages	Plastic	Cytochemical staining	[8, 31, 106, 136, 137]
			Bulk population		Plastic	Enzymatic evaluation	[106]
			Bulk population		Plastic	Gene evaluation	[8, 31, 106, 136, 137]
		2nd trim, term	Bulk population		Plastic	Cytochemical staining	[123]
		1st trim	Bulk population		Plastic	Cytochemical staining, enzymatic evaluation	[138]
Unspecified placental tissue and/or a mix of placental tissues		Term	Bulk population	Cultured passages	Plastic	Cytochemical staining	[40, 41, 58, 59, 139–152]
					Plastic	Immunostaining	[41]
					Plastic	Enzymatic evaluation	[40, 59, 143, 152]
					Plastic	Gene evaluation	[40, 41, 59, 144, 151, 152]
					[PAH/PSS]_9_ PEM coated ITO treated glass electrodes	Cytochemical staining	[153]
					Nano-biphasic calcium phosphate ceramics	Cytochemical staining, gene evaluation	[105]
					PCL-PEG-PCL scaffold	Cytochemical staining, enzymatic and protein evaluation	[77]
			FZD9^+^CD10^+^CD26^+^		Plastic	Cytochemical staining, enzymatic and gene evaluation	[35]
			AdLMP-1 or AdLacZ transduced cells		Plastic	Cytochemical staining, gene and protein evaluation	[154]

*NS* not specified; *trim* trimester; *PU* polyurethane; *PEM* polyelectrolyte multilayers; *PCL* polycaprolactone; *PEG* poly ethylene glycol; PAH poly (allylamine hydrochloride); *PSS* poly(styrene sulfonate); *ITO* indium tin oxide; *FZD9* frizzled-9

**Table 2:** Summary of studies describing in vitro osteogenic differentiation of term hUC-MSC and hWJ-MSC at passages

MSC lineages used	Growth support	Readout	References
Bulk population	Plastic	Cytochemical staining	[17, 20, 47, 52, 55, 57, 61, 64, 76, 92, 96, 98, 155–179]*
		Immunostaining	[52, 57, 96, 156, 175, 180]
		Enzymatic evaluation	[17, 52, 57, 61, 76, 96, 98, 157–159, 162, 173, 177, 180–183]**
		Gene evaluation	[17, 20, 57, 61, 64, 92, 98, 155, 158–160, 162, 164, 165, 167, 171, 173, 175, 177, 180, 184]*
		Protein evaluation	[57, 98, 184]
		Biochemical assay	[17, 76, 170, 173]
	Rod-like-nHA and flake-like-micro-HA coatings on Mg-Zn-Ca alloy substrates	Cytochemical and immunostaining, enzymatic and gene evaluation	[185]
	Type I atelocollagen-coated Bioflex® plate stimulated with Flexcell system	Gene and protein evaluation	[63]
	Fibronectin coating	Cytochemical and immunostaining, enzymatic, gene and protein evaluation	[186]
		Cytochemical and immunostaining, electron microscopy	[187]
	Collagen I coating	Cytochemical staining, protein evaluation	[65]
	PAAM substrates	Cytochemical staining, gene evaluation	[188]
	Porcine urinary bladder matrix scaffold	Cytochemical and immunostaining, enzymatic and gene evaluation, electron microscopy, spectroscopy	[69]
	Collagen scaffold	Cytochemical staining, enzymatic evaluation	[189]
		Cytochemical and immunostaining, gene evaluation and electron microscopy	[68]
	PGA and derivatives scaffold	Cytochemical staining, enzymatic and gene evaluation	[79, 81]
		Protein evaluation	[81]
		Biochemical assay	[78, 79]
		Electron microscopy	[79]
	PCL and derivatives scaffold	Cytochemical staining, enzymatic and gene evaluation, electron microscopy	[75, 76]
		Immunostaining	[75]
		Spectroscopy	[76]
	PLLA scaffold	Cytochemical staining, protein and gene evaluation	[82, 83]
		Immunostaining	[82]
	Nano-biphasic calcium phosphate ceramics	Cytochemical staining, gene evaluation	[105]
	45S5 Bioglass-based scaffold	Cytochemical staining, protein evaluation	[190]
	CPC scaffold and derivatives	Cytochemical staining	[45, 46, 84, 85, 87, 89–91, 93–95, 109]
		Enzymatic evaluation	[45, 46, 88–91]
		Gene evaluation	[45, 46, 85–89, 91, 93–95, 109]
		Electron microscopy	[86, 88, 89]
		Spectroscopy	[88, 89]
Bulk population and HSP90β transfected cells	Plastic	Cytochemical staining, enzymatic and gene evaluation	[108]
Lentiviral-transduced cells	Plastic	Enzymatic and gene evaluation	[191]
Osx-transfected MSC	Plastic	Enzymatic and gene evaluation	[97]

*PCL* polycaprolactone; *PAAM* polyacrylamide hydrogel; *PGA* polyglycolic acid; *PLLA* poly(L-Lactic acid); *CPC* calcium phosphate cement; *nHA* nano-hydroxyapatite; *Osx* osterix

* Reference 92: hUC-MSC were encapsulated within alginate microbeads, oxidized alginate microbeads, and oxidized alginate-fibrin microbeads

** Reference 182: UCX® cells: human stem cells derived from the umbilical cord tissue (Wharton’s jelly)

**Table 3:** Summary of studies describing in vivo osteogenic differentiation of MSC derived from fetal and maternal tissues

	Placental region	Gestation time	MSC lineages used	Cell pre-treatment	Growth support	Site of implantation	Animal model	Readout	References
Fetal	Chorion	Term	CD31^−^/CD45^−^; CD31^−^/CD45^−^/CD349^+^ and CD31^−^/CD45^−^/CD349^−^	Plated on scaffold 2 h before implantation	Gelform	Local to bone defect	C57/BL6 mice with induced femur fracture	Histological staining, radiography	[107]
		1st trim, term	Bulk population	None	None	Intraperitoneal	Heterozygous male and female (B6C3Fe a/a-Col1a2^oim^/Col1a2^oim^) mice	Mechanical tests	[111]
		1st trim	Bulk population	None	None	Intraperitoneal	Heterozygous male and female (B6C3Fe a/a-Col1a2^oim^/Col1a2^oim^) mice	Immunostaining, gene and protein evaluation, radiography, mechanical tests, histomorphometry	[112]
	Umbilical cord or Wharton’s jelly	Term	Bulk population	None	nHA/Coll/PLA scaffold	Subcutaneous	Nude mice	Immunostaining, electron microscopy	[96]
		Term	Bulk population	2 wks in OM	PCL-TCP scaffold	Subcutaneous	NOD/SCID mice	Immunohistochemistry, micro-CT	[76]
		Term	Bulk population	None	Fibrin glue prepared using human thrombin	Local to bone defect	SCID mice with segmental femur defect	Immunohistochemistry, radiography, mammography	[108]
		Term	Bulk population	1 wk in CM + 3 wks in OM + rhBMP	HA scaffold	Subcutaneous	Nude mice	Immunostaining, gene evaluation	[55]
		NS	Bulk population	None	None	Local to bone defect	Sprague Dawley rats with tibial non-union fracture	Immunohistochemistry radiography	[110]
		Term	Bulk population	1wk in CM + 3 wks in OM + UCB-PRP (1st medium change)	UCB-fibrin scaffold	Subcutaneous	Nude mice	Immunohistochemistry, evaluation, electron microscopy, spectroscopy, micro-CT	[56]
		NS	Bulk population	14 days in OM	RGD-grafted CPC scaffold	Local to bone defect	Nude rats with critical-size cranial defect	Histological staining, histomorphological analysis, micro-CT	[109]
		NS	Bulk population	None or 4 days in OM	Triosite scaffold	Local to bone defect	Nude rats with critical-size femur defect	Histological staining, radiography, micro-CT	[102]
		Term	Osx-transfected MSC	None	PLGA scaffold	Subcutaneous	Nude mice	Histological staining, gene evaluation, radiography	[97]
		NS	Bulk population	7 days in OM	Dentine disk + fibrin sealant gum	Subcutaneous	BALB/c-nu with SCID	Immunohistochemistry	[98]
		Term	Bulk population	Constructs cultured for 14 days	PLGA, nHA/PLGA, CS/PLGA, nHA/CS/PLGA scaffolds	Subcutaneous	Nude mice	Histological staining	[79]
Maternal	Decidua	Term	Bulk population	24 h in CM + 4 days in perfusion culture system	PLGA scaffold	Local to bone defect	Sprague Dawley rats with induced cranial defect	Histological staining	[106]
Unspecified placental tissue		NS	Bulk population	None	Nano-biphasic CaP ceramics	Local to bone defect	Wistar rats with induced femur fracture	Immunohistochemistry, radiography	[105]

*NS* not specified; *trim* trimester; *Osx* osterix; *OM* osteogenic medium; *CM* culture medium; polycaprolactone (PCL)- tricalcium phosphate (TCP); *PLA* poly(lactic acid); *UCB-PRP* umbilical cord blood derived platelet rich plasma; *RGD* arginylglycyl-aspartic acid; *CPC* calcium phosphate cement; *PLGA* poly(lactide-co-glycolide); *nHA* nano-hydroxyapatite; *CS* chitosan.

### In Vitro Two-Dimensional Osteogenic Differentiation

In this section we will focus on data describing in vitro two-dimensional osteogenic differentiation, and approaches to improve the outcomes, such as selection of subpopulations and modification of culture conditions, and the results obtained.

#### Selection of Subpopulations

Considering the heterogeneity of the mesenchymal stromal population within the placental regions, some authors attempted to select for subpopulations more prone to differentiate towards the osteogenic lineage (Table 1). For example, positive selection using Frizzled-9 (FDZ9), as based on information available on BM-MSC [34], together with CD10 and CD26 resulted in a subpopulation of hAMSC with elevated expression levels of osteocalcin (OC), increased number of cells with positive alkaline phosphatase (ALP) activity, and higher number of calcium-rich nodules in comparison with unselected cells, altogether suggesting increased differentiation potential of the selected cells [35]. On the other hand, decreased osteogenic differentiation was observed when hAMSC and hCMSC were selected based upon CD105 positivity [36]. Moreover, a CD44^+^/CD73^+^/CD105^−^ subpopulation of hAMSC showed higher bone matrix mineralization and stronger expression of secreted protein acidic and rich in cysteine (SPARC) and osteopontin (OPN), two markers commonly associated to mineralization, than CD44^+^/CD73^+^/CD105^+^ cells, while no significant differences were observed for collagen type I-alpha 1 (COLIA1) and OC expression [15]. CD271 has also been investigated as a potential selection marker for MSC selection. Previously, selection for CD271 positivity was shown to identify a BM-MSC subpopulation more prone to differentiate into the osteogenic lineage [37]. More recently, our group applied this same selection in hAMSC and hCMSC populations, where clearly enhanced osteogenic differentiation in the CD271-enriched fractions were observed [10]. It was also reported that side-population derived from hAMSC showed osteogenic differentiation potential [38], and that both CD106^+^ and CD106^−^ fractions from hCMSC demonstrated similar ability to differentiate into the osteogenic lineage [39].

As previously mentioned, hPD-MSC share the majority of features with BM-MSC, however some differences have been reported. For example, increased expression of CD146 on BM-MSC in comparison to hPD-MSC, together with that of ALP assessed before differentiation, has been shown to correlate with improved osteogenic differentiation capacity [40].

Transcriptomic analysis of hPD-MSC showed differential expression of osteogenesis related genes, assessed either prior to (Runx2, Twist2), or after (BMP2, osteomodulin, SFRP1, SFRP4) osteoinduction and differentiation [41].

#### Composition of Differentiation Media

Attempts to enhance osteogenic differentiation have been made by modifying the composition of the osteogenic-inductive medium (i.e. different supplements) [*reviewed in* [42, 43]]. Osteogenic differentiation medium is generally composed of basal medium (e.g. DMEM) supplemented with 10 % FBS, dexamethasone, β-glycerol phosphate, ascorbic acid, 1α,25-dihydroxyvitamin D3 [18, 44–46]. Osteogenic differentiation of hWJ-MSC was observed at early and late passages (15–20) after culturing in either DMEM low glucose, DMEM high glucose, DMEM/F12 or DMEM-KO as a basal media, with the latter inducing even higher calcification [47]. FBS has been investigated as a factor which could influence osteogenic potential. FBS removal from the culture media was found to result in the absence of mineralization in hAMSC, hCMSC, BM-MSC, and adipose tissue-derived stromal cells (ASC) [48]. Accordingly, the presence of FBS has been shown to enhance osteogenic-associated marker ALP and bone sialoprotein (BSP) expression in BM-MSC and ASC, while low or no expression was found in fetal-derived MSC in both culture conditions [48]. Regarding the use of xenobiotic-free medium for a more clinically-relevant approach, some studies have also investigated the use of platelet-rich plasma (PRP) [49], platelet lysate [50–54] or platelet concentrate [40] as a culture media supplement. UCB-derived PRP has also been used as a growth factor to assist in vivo osteogenic differentiation [55, 56].

Other supplements have been used in the attempt to boost osteogenic differentiation, such as osteogenic proteins (rhBMP-2) [57], osteoactivin [58], and chemical entities such as bortezomib [59] and 5-aminoimidazole-4-carboxamide-1-β-riboside (AICAR) [60]. Moreover, the inflammatory microenvironment, common to degenerative diseases in which MSC are used as cell-therapy, has also been investigated for its impact on osteogenic differentiation. For instance, pretreatment of hWJ-MSC with an inflammatory cytokine cocktail (IL-1β, IFN-γ, TNF-α and IFN-α) increased differentiation into osteoblasts similarly to BM-MSC and ASC, although in absence of stimulation with inflammatory cytokines, the osteogenic differentiation capability of hWJ-MSC was lower than that of BM-MSC and ASC [61]. Moreover, TLR3 and TLR4 ligation did not affect hWJ-MSC, while it enhanced ASC osteogenic potential [61].

#### Mechanical Stimulation

Mechanical stimulation mimicking the movement presented in in vivo settings has been reported as another approach to enhance in vitro MSC osteogenic differentiation. Cyclic uniaxial stimulation, one of the most widely used mechanical stimulations in vitro, was shown to accelerate osteogenic differentiation of BM-MSC [62]. Mechanical stimulation provided by the Flexcell system, potentiated the osteogenic differentiation of hUC-MSC (in the presence of osteogenic supplements), as indicated by an increase of the osteogenic gene (osteoprotegerin, OC, OPN, osteonectin, collagen I (Coll I), Coll III and vimentin), and protein (BSP and vimentin) expression [63].

In conclusion, differentiation toward the osteogenic lineage is evident in the majority of in vitro studies, however there are reports showing weak [36, 40, 41, 48, 61] or even lack of osteogenic differentiation [64, 65].

### In Vitro Three-Dimensional Osteogenic Differentiation

The number of studies investigating three-dimensional environment for bone tissue formation is constantly increasing. So far, most of the studies present in literature investigate hWJ-MSC rather than fetal-membrane derived MSC, suggesting a growing interest in this specific placental cell population. In this section, we will present data describing the osteogenic differentiation on different types of three-dimensional constructs, namely non-synthetic, polymer-based, and ceramic scaffolds (see also Tables 1 and 2).

#### Osteogenic Differentiation on Non-Synthetic Scaffolds

Generally, synthetic biomaterials are employed for the preparation of scaffolds to support osteogenic differentiation, although some groups have attempted to use non-synthetic biomaterials in order to enhance biocompatibility and biodegradability. For example, commonly used non-synthetic scaffolds are collagens, which are abundant in the osteocyte environment, have high mechanical strength, and have been shown to stimulate MSC to differentiate into osteoblast-like cells, altogether initiating new bone formation [66, 67]. Cultivation of hUC-MSC, and BM-MSC, on a collagen I/III gel has been shown to lead to deposition of hydroxyapatite (HA)/calcium crystals and to an active shift of the collagen I/III ratio in favor of collagen I, the main component of bone extracellular matrix (ECM). Moreover, production of other ECM proteins like collagen IV, laminin and glycosaminoglycans (GAGs), was also observed in a manner comparable to functional osteocytes and osteoblasts [68].

Natural biomaterials have also been shown to be able to support proliferation and osteogenic differentiation of hPD-MSC [69, 70]. Evidence of osteogenic differentiation, as suggested by higher gene expression of Runx2, OPN, Coll I, ALP activity and mineralized matrix deposition, were provided when hWJ-MSC were seeded on an extracellular matrix scaffold (porcine urinary bladder derived matrix) in the presence of differentiation medium [69]. Moreover, osteoinductive effects of Nukbone® (NBK), a human bone biomimetic material from bovine bone matrix, were observed in hAMSC without the application of differentiation medium, as demonstrated by OC and Runx2 gene expression [70]. Interestingly, human chorionic membrane has also been used as cell-free extracellular matrix scaffold for osteogenic differentiation. Up-regulation of OC and OPN gene expression, and positive immunohistochemical staining for OC, OPN and Runx2, but not Coll I, together with increased Ca^2+^ concentrations, characteristic for advanced mineralization, have been documented [71]. The amniotic membrane, which has been largely used in the field of tissue engineering as a biological scaffold [*reviewed in* [72] and [73]], has been recently demonstrated to act as a natural cell substrate for the osteogenic differentiation of amniotic membrane-derived cells without cell isolation, leaving cells residing within their natural environment [74].

#### Osteogenic Differentiation on Polymer-Based Scaffolds

Osteogenic differentiation of hPD-MSC has been widely assessed on scaffolds containing polycaprolactone (PCL) and/or its derivatives [75–77]. Mineralization and up-regulation of osteogenic genes were observed in MSC derived from umbilical cord when collagen and HA [75] or β-tricalcium phosphate (TCP) [76] were added to PCL scaffold, even though hUC-MSC demonstrated lower osteogenic differentiation potential than fetal BM-MSC [76]. Culture of hPD-MSC on PCEC (PCL-poly(ethylene glycol)(PEG)-PCL) copolymer revealed osteoblast differentiation as demonstrated by mineral deposition, expression of OPN and OC, and increased ALP activity compared to hPD-MSC differentiated on plastic plates [77]. Mineralization and osteogenic gene expression were also observed on hUC-MSC seeded on polyglycolic acid (PGA) and derivatives (poly(lactide-co-glycolide), PLGA) scaffolds [78, 79]. Greater ALP and OC expression, together with higher ALP activity and OC content of hUC-MSC cultured on nanoHA/chitosan(CS)/PLGA scaffold was observed, as compared to PLGA, nanoHA/PLGA and CS/PLGA [79]. Polyurethanes (PU) are another example of polymers applied for supporting osteogenic differentiation, however, only a small amount of calcium deposition has been observed when chorion-MSC were cultured on PU foam with induction medium [80].

Some strategies have been designed for osteochondral tissue engineering in order to simultaneously obtain osteo- and chondrogenic differentiation on the same construct [81, 82]. For example, recently, an in vitro study reported that microsphere-based scaffolds constructed to release TGF-β1 and BMP-2 (factors known for inducing chondrogenesis and osteogenesis, respectively), with a gradual and continuous transition in the release of TGF-β1 and BMP-2 from one side of the scaffold to the other, was able to promote osteogenic differentiation of hUC-MSC and BM-MSC [81]. A significant increase was observed in cell number, GAGs and collagen content, and ALP activity in the gradient scaffold [81]. The ability of poly(L-Lactic acid) (PLLA) scaffolds to support osteogenic differentiation of hUC-MSC has been previously demonstrated [83]. In a follow-up study by the same group, a further strategy was implemented by sandwiching hUC-MSC between chondrogenic and osteogenic PLLA scaffolds and then suturing them together [82]. Osteochondral composites with hUC-MSC exhibited the best integration and transition of ECM between the layers, while composites without cells presented better distribution and stronger staining of calcium, Coll II and aggrecan [82].

#### Osteogenic Differentiation on Ceramic Scaffolds

The use of ceramic scaffolds as support for osteogenic differentiation is widely recognized. For the most part, hPD-MSC have been investigated with calcium phosphate cement (CPC)-based scaffolds, since this type of scaffold has been shown to be an injectable and resorbable bioceramic. Using hWJ-MSC, efforts have been made to enhance physical and mechanical properties [45, 84–87], cell distribution within the scaffold [46, 88–92], cell adhesion to the scaffold surface [46, 84, 93–95], and in vitro osteogenic differentiation.

hUC-MSC have been seeded on traditional CPC, and attachment, proliferation and differentiation toward the osteogenic lineage have been demonstrated [86], and even improved differentiation when CPC was supplemented with different biofunctional agents (fibronectin, fibronectin-like engineered polymer protein (FEPP), arginylglycyl-aspartic acid (RGD), Geltrex, platelet concentrate), fibers (collagen, PLGA fibers) [84, 93, 94], or mannitol [45]. In addition, alginate hydrogel beads have been used to protect hUC-MSC against mixing and injection forces and to favor the distribution within the scaffold, while maintaining the osteogenic differentiation capabilities of cells [86, 88, 89, 91]. Moreover, as the cells are encapsulated within the microbeads, rapid microbead degradation followed by cell release is required. Improvement of the rate of microbead degradation, in accordance with the release of hUC-MSC, has been developed maintaining cell differentiation potential toward the osteogenic lineage [46, 92, 95]. Indeed, released hUC-MSC underwent osteogenic differentiation as indicated by up-regulation of osteogenic gene expression (ALP, OC, Coll I), ALP activity and mineral synthesis [46, 95]. Interestingly, improvement of osteogenic differentiation was achieved by encapsulation of pre-differentiated hUC-MSC, or by delivery of osteogenic medium, instead of BMP-2, into the microbeads together with hUC-MSC. The results showed that each of these approaches allowed hUC-MSC to be successfully differentiated into the osteogenic lineage [90].

## In Vivo Osteogenic Potential

Currently, the number of studies describing in vivo applications of hPD-MSC for bone defect restoration is increasing (Table 3). Proof of principle studies of the in vivo osteogenic potential of hPD-MSC may be achieved using subcutaneous injection of experimental constructs to induce ectopic bone formation and to assess the feasibility of different biomaterials. For example, different types of constructs supporting osteogenic differentiation, such as nano-HA/CS/poly(lactide-co-glycolide) (nHA/CS/PLGA), have been investigated using hUC-MSC. The application of nHA/collagen/PLA, nHA/PLGA, CS/PLGA, or nHA/CS/PLGA seeded with hUC-MSC indicated immature bone tissue formation after subcutaneous implantation into nude mice [55, 79, 96]. Enhanced bone formation was observed on PLGA scaffolds also when osterix-transfected hUC-MSC were used, a transcription factor known for its role in osteoblast differentiation and bone formation, together with up-regulated mRNA expression of ALP, OC, OPN and Coll I when compared to non-transfected hUC-MSC/PLGA and mock-hUC-MSC/PLGA controls [97]. In vivo subcutaneous implantation of MSC from different sources on PCL-TCP scaffolds has been shown to result in superior osteogenic potential of fetal BM-MSC, when compared to adult BM-MSC, perinatal hUC-MSC, and ASC [76].

In addition, considering the clinical application of MSC, the use of autologous tissues to avoid/minimize the probability of infections and immune response to biomaterials has been investigated. Recently, umbilical cord blood (UCB)-derived fibrin as a scaffold for hUC-MSC, UCB-derived platelet-rich-plasma (UCB-PRP) as a source of growth factors, and UCB-derived serum for hUC-MSC culture were studied in vivo after subcutaneous implantation. Although no defined bone tissue in vivo was seen, ectopic calcification was observed [56]. Application of hUC-MSC have also been studied in dental regeneration in terms of periodontal tissue healing, important to increase success rate of autotransplantation of teeth. In vivo osteogenic differentiation of hUC-MSC on dentine disc showed the ability to form cementum-like deposits after subcutaneous implantation suggesting that hUC-MSC may be useful in this field [98].

### hPD-MSC for Regeneration of Bone Defects

Most of the studies regarding in vivo bone regeneration investigate femur or cranial fractures. Critical-size calvarial defects are widely employed to study bone healing in animal models, mostly rodents, because the calvaria are large plates that facilitate the creation of defects, implantation of grafts and the analysis (histology, imaging) of reconstruction [99, 100]. Segmental defects in long bones are also widely used as clinically relevant models [101–104].

The number of studies investigating hPD-MSC in femur fractures is increasing. Femur defects have been treated with hPD-MSC used in combination with nanosized biphasic Ca/P ceramics and led to complete recovery of the defect with no graft rejection or inflammation process [105]. Good integration of the material with surrounding host tissues and newly formed bone was observed, together with expression of human Runx2 mRNA, indicating that implanted MSC were able to survive and promote in vivo osteogenesis [105].

In vitro attempts have been made to select for a subpopulation more prone to differentiate into osteogenic lineage in in vivo models. Using a method based on time-gradient attachment, hDMSC seeded into PLGA scaffolds have shown significant bone formation 20 weeks after transplantation into full-thickness calvaria defects, as confirmed by histological and immunohistochemical assays [106]. Transplantation of both FZD9 (CD349)-positive and -negative hCMSC in femur defects in mice resulted in facilitating new bone calcification in fractured femurs [107].

In vitro knock down of HSP90β (a cell proliferation related protein) on hWJ-MSC revealed enhanced osteogenic differentiation. Following experiments in in vivo segmental bone defects resulted in more prominent bone tissue in comparison to hWJ-MSC without HSP90β knockdown [108].

The in vivo osteogenic potential of hUC-MSC has been compared to adult MSC from BM [109] and adipose tissue, and perinatal UCB [102] using critical-sized cranial [109] and femoral defect models in rat [102]. Based on quantitative assessment, the authors concluded that there were no significant differences between fetal and adult MSC [102, 109].

Treatment of non-union fractures has rarely been investigated. Transplantation for non-union fractures in a rat model using hUC-MSC in presence of blood plasma resulted in new bone formation and disappearing cortical gaps, suggesting a possible role of hUC-MSC in bone healing [110].

### hPD-MSC for the Treatment of Bone-Related Diseases

hPD-MSC have also been investigated in vivo in other bone-related diseases, such as osteogenesis imperfecta (OI) and multiple myeloma (MM).

In a comparison study investigating the characteristics of hCMSC obtained from first trimester with those obtained from term placenta, intraperitoneal injection of early (first trimester) hCMSC in a murine model of OI (*oim*) was more efficient in improving overall bone quality, as shown by a reduction of fractures, increased bone volume and bone plasticity, when compared to term hCMSC [111]. Moreover, transplanted first trimester hCMSC underwent differentiation into functional osteoblasts as confirmed by COL1A2 protein expression in the femoral bones of *oim* [112].

Multiple myeloma is a malignancy giving rise to osteolytic bone disease and increased fractures. An interesting study has shown that hPD-MSC were able to suppress MM-induced bone lesions, and also tumor growth in bone, through osteoclast formation and stimulation of endogenous osteoblastogenesis when injected into myelomatous osteolytic lesions [113].

## Conclusions

In conclusion, the majority of in vitro studies support the potential of hPD-MSC to differentiate into the osteogenic lineage, suggesting their possible use in regenerative medicine to repair osteo-related defects. A large amount of evidence has been provided based on in vitro calcium deposition and/or gene expression, each of which point toward different stages of differentiation potential. In vivo studies, even if in some cases are very encouraging, are still somewhat preliminary and are mostly based on the use of one region of placenta, namely Wharton’s jelly.

Hence, in order to apply hPD-MSC for bone regeneration, further investigations should be focused on the selection of the most prone subpopulation, pre-committing hPD-MSC prior to in vivo use, and selection of the most appropriate support for differentiation.
